# The Relationship Between Iron Status and Atherosclerotic Cardiovascular Disease Risk in Non-anemic Patients Without a History of Cardiovascular Diseases: A Cross-Sectional Study

**DOI:** 10.7759/cureus.29439

**Published:** 2022-09-22

**Authors:** Tomasz Skrzypczak, Anna Skrzypczak, Jakub Michałowicz

**Affiliations:** 1 Faculty of Medicine, Wroclaw Medical University, Wroclaw, POL; 2 Faculty of Dentistry, Wroclaw Medical University, Wroclaw, POL

**Keywords:** cardiovascular risk score, nhanes data, national health and nutrition examination survey (nhanes), ascvd risk, non-anemic iron deficiency (naid)

## Abstract

Iron deficiency (ID) and iron status in non-anemic patients with atherosclerotic cardiovascular disease (ASCVD) risk without a history of cardiovascular diseases is still weakly explored. In this study, the authors evaluated the most common ID definitions in this group of patients. A total of 533 participants from the National Health and Nutrition Examination Survey (NHANES) were collected from 2005-2006, 2017-2018, and 2017-2020 records. Participants were divided according to their ASCVD risk score to the following groups: low (n=168, 32%), borderline (n=43, 8%), intermediate (n=200, 37%), and high (n=122, 23%). There was a higher prevalence of ID in low- and borderline-risk groups in contrast to intermediate- and high-risk groups. Higher serum ferritin concentrations were observed in groups with a greater ASCVD risk score. Transferrin saturation (TSAT) was comparable in all ASCVD categories. Lack of ID, defined by three different guidelines that are mainly based on serum ferritin levels, predisposed to a higher ASCVD risk category. Normal iron status, defined by these three guidelines, was positively associated with the male gender. The opposite association was observed for non-Hispanic Whites. The analyzed criteria of ID, based mostly on serum ferritin levels, demonstrated limited usefulness in patients with increased ASCVD risk. Further studies should be done to determine proper ID diagnostic criteria in non-anemic patients without a previous cardiovascular history with elevated ASCVD risk.

## Introduction

Iron deficiency (ID) has become recognized as an independent entity beyond anemia with good prognostic value in diseased cohorts, mainly cardiovascular ones [1,2]. Since Sullivan proposed the iron-heart hypothesis in 1981 [3], the relationship between iron status and cardiovascular disease has been investigated extensively [4]. This suggested a positive association between iron deposits and the risk of cardiovascular diseases. Several prospective studies supported this theory, showing that elevated serum ferritin concentrations were associated with a higher risk of myocardial infarction [5] and carotid atherosclerosis [6]. Many epidemiological investigations did not confirm this correlation [7-9]. Furthermore, some studies revealed that low iron status could be an independent risk factor for coronary disease and stroke [10,11].

While the impact of elevated serum ferritin levels on cardiovascular risk remains not clearly understood [1,7,9], the relationship between iron deficiency (ID) and increased cardiovascular mortality has been proven in large prospective studies [1,7]. Most of the current ID definitions utilize the serum ferritin threshold as a criterion [1]. The impact of serum ferritin fluctuations on ID diagnosis in patients with heart failure (HF) has been recently heavily investigated [12]. ID in patients with HF and the presence or absence of anemia was associated with poorer quality of life, exercise capacity, and prognosis [12-16]. Also, in the general population, ID was associated with increased all-cause mortality in the mid to long term [1]. The recent ID criteria were mostly established for the purpose of clinical trials [12]. Their diagnostic utility for patients with HF was questioned [12]. To date, these criteria have been not evaluated on non-anemic patients with increased atherosclerotic cardiovascular disease (ASCVD) risk without cardiovascular history [12-16]. The authors aimed to evaluate the most often utilized ID definitions on the non-anemic patient population without cardiovascular history classified according to ASCVD risk categories.

## Materials and methods

The association between iron status and ASCVD risk was investigated using a 10-year ASCVD risk score and the National Health and Nutrition Examination Survey (NHANES).

The NHANES is a review of the US (United States) population, conducted through a series of examinations and interviews at independent research centers [17]. In this study, the following NHANES records were utilized: 2005-2006, 2017-2018, and 2017-2020. Participants who fall within any of the following criteria were excluded from the analysis: age < 20; hemoglobin (Hb) < 12 g/dL in women and Hb < 13 g/dL in men; serum C-reactive protein (CRP) > 5 mg/dL; race other than non-Hispanic White or non-Hispanic Black; and history of congestive heart failure, coronary heart disease, angina pectoris, myocardial infarction, and stroke. Those who missed any analyzed variable were excluded from the study. History of cardiovascular diseases, tobacco smoking status, race, gender, and age were self-reported by NHANES participants. When NHANES reported high-sensitivity CRP, data were transformed into CRP levels according to a previously validated method [18].

The ASCVD risk score was calculated in accordance with the Revised Pooled Cohort Equations for Estimating Atherosclerotic Cardiovascular Disease Risk [19], an updated equation for the American College of Cardiology/American Heart Association (ACC/AHA) 10-year risk score [19,20]. The risk was reported as percentages. Participants were classified according to the 2013 ACC/AHA Guideline on the Assessment of Cardiovascular Risk [20] into the following ASCVD categories: low (<5%), borderline (5%-7.4%), intermediate (7.5%-19.9%), and high (20%) [20].

The following definitions were employed in this study: diabetes mellitus was present if hemoglobin A1c $\geq$ 6.5%; hypertension was diagnosed when systolic blood pressure (BP) $\geq$ 140 mmHg or diastolic BP $\geq$ 90 mmHg or antihypertensive mediation was used by the participant, in accordance to the 2020 International Society of Hypertension Global Hypertension Practice Guidelines [21]; and obesity was diagnosed when body mass index $\geq$ 30 kg/m^2^.

For the present analysis, the following definitions of iron deficiency were used: functional iron deficiency (FID) (1), ferritin levels < 100 ng/mL; FID (2), ferritin levels between 100 and 299 ng/mL, if the transferrin saturation (TSAT) is below 20%, both recommended in patients with heart failure [16,22]; absolute iron deficiency (AID), serum ferritin levels < 30 ng/mL (this definition is common in internal medicine community) [23]; World Health Organization iron deficiency (WHO ID), serum ferritin < 70 ng/mL, recommended for individuals with infection or inflammation, with CRP levels < 5 mg/L [24]; and iron deficiency without anemia (IDWA), diagnosed with the algorithm proposed by Al-Naseem et al. [25], with hypertension, diabetes mellitus, and obesity classified as chronic diseases.

Participant characteristics were compared according to the ASCVD risk category. Categorical variables were presented as frequency and percentage. The chi-square test was used to compare categorical variables. Continuous variables were presented as mean ± standard deviation (SD). Continuous variables were examined using the Kolmogorov-Smirnov and Shapiro-Wilk tests for normality. Then, univariate analyses (Mann-Whitney U and Kruskal-Wallis tests) were executed to assess the relationships between the analyzed variables and the ASCVD risk category.

Multinominal logistic regression analysis (MLRA) was performed to examine the impact of iron deficiency on ASCVD risk. Independent variables were the presence and absence of ID that differed statistically significant between ASCVD risk categories in univariate analysis. Dependent variables were ASCVD risk group categories with the low-risk group always used as a reference. In addition, MLRA was performed to investigate potential confounding variables on participant iron status. Variables that significantly differed between ASCVD risk groups in univariate analyses served as independent in this MLRA. Dependent variables also include ID diagnosed with included definitions with patient normal iron status always used as a reference. In all these MLRA, the odds ratio (OR) with 95% confidence interval (95%CI) was calculated.

All statistical analyses were performed using IBM Statistical Package for the Social Sciences (SPSS) Statistics version 1.0.0.1347 (IBM Corp., Armonk, NY, USA). P<0.05 was considered statistically significant.

## Results

In this analysis, there were a total of 533 participants in all ASCVD risk groups after exclusions. Population characteristics stratified by ASCVD risk categories are shown in Table 1.

**Table 1:** Population characteristics. *P<0.05 Data are reported as mean ± standard deviation (SD). CRP: C-reactive protein (patients with CRP > 5 mg/dL were excluded from the study); HDL: high-density lipoprotein; ASCVD: atherosclerotic cardiovascular disease; TSAT: transferrin saturation; low: ASCVD score < 5%; borderline: ASCVD score between 5% and 7.4%; intermediate: ASCVD score between 7.5% and 19.9%; high: ASCVD score > 20%

	ASCVD risk category			
Characteristics	Low (n=168)	Borderline (n=43)	Intermediate (n=200)	High (n=122)
Age	46±1	57±1	65±1	71±1
Men*	46 (27%)	22 (51%)	142 (71%)	93 (76%)
Non-Hispanic White*	119 (71%)	23 (54%)	122 (61%)	62 (51%)
Non-Hispanic Black*	49 (29%)	20 (46%)	78 (39%)	60 (49%)
Hypertension*	105 (63%)	32 (74.4%)	150 (75%)	103 (84%)
Obesity	87 (52%)	20 (47%)	88 (44%)	54 (44%)
Diabetes mellitus*	2 (1%)	3 (7%)	20 (10%)	53 (43%)
Current tobacco smoker	69 (41%)	17 (40%)	77 (39%)	52 (43%)
Total cholesterol* (mg/dL)	191±3	203±7	184±3	186±4
HDL-C* (mg/dL)	59±2	56±3	54±1	48±1
Ferritin* (ng/mL)	144.7±9.8	149.8±18.2	195.5±11.6	193.7±13.5
TSAT (%)	28.8±1	28.4±1.6	29.6±0.7	29.4±0.9

**Figure 1:**
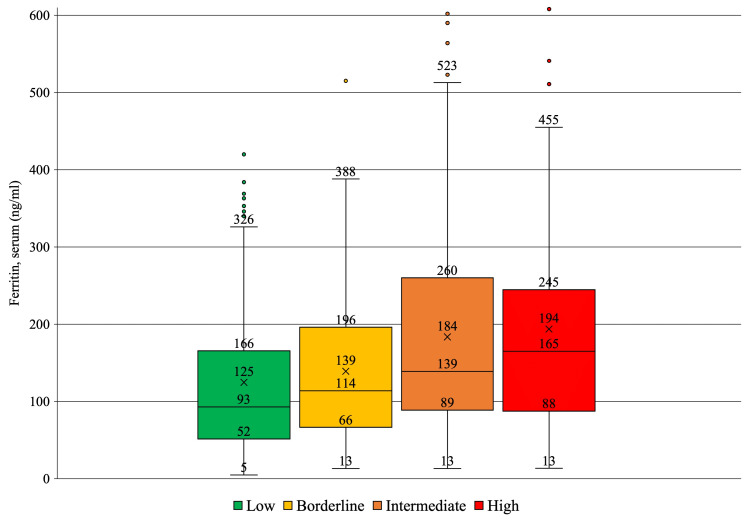
Ferritin levels in ASCVD risk groups. The differences between groups were statistically significant (P<0.001). ASCVD: atherosclerotic cardiovascular disease; low: ASCVD score < 5%; borderline: ASCVD score between 5% and 7.4%; intermediate: ASCVD score between 7.5% and 19.9%; high: ASCVD score > 20%

Non-Hispanic White participants and men were more prevalent in groups classified with higher ASCVD risk. Hypertension and diabetes mellitus were more common in groups with elevated ASCVD risk. Serum ferritin concentrations were significantly higher in groups with a greater ASCVD risk score. The association is presented in Figure 1.

The prevalence of ID among ASCVD risk groups, stratified by ID definition, is presented in Table 2. WHO ID, FID (1), and IDWA were statistically more common in low and borderline categories than intermediate and high. There were no statistically significant differences between groups in AID and FID (2) prevalence.

**Table 2:** Iron deficiency in non-anemic patients classified by ASCVD risk score. *P<0.05 (calculated using the chi-square test) ID: iron deficiency; AID: absolute iron deficiency; WHO ID: World Health Organization iron deficiency; FID (1): functional iron deficiency (1); FID (2): functional iron deficiency (2); IDWA: iron deficiency without anemia; ASCVD: atherosclerotic cardiovascular disease; low: ASCVD score < 5%; borderline: ASCVD score between 5% and 7.4%; intermediate: ASCVD score between 7.5% and 19.9%; high: ASCVD score > 20%

ID definition	Overall (N=533)	ASCVD risk category			
		Low (n=168)	Borderline (n=43)	Intermediate (n=200)	High (n=122)
AID	37 (7%)	18 (11%)	3 (7%)	10 (5%)	6 (5%)
WHO ID*	121 (23%)	54 (32%)	14 (33%)	32 (16%)	21 (17%)
FID (1)*	198 (37%)	85 (51%)	19 (44%)	59 (30%)	35 (29%)
FID (2)	10 (2%)	2 (1%)	0 (0%)	4 (2%)	4 (3%)
IDWA*	212 (40%)	83 (49%)	19 (44%)	67 (34%)	43 (35%)

The results of MLRA regression between the presence/absence of ID and ASCVD risk category are shown in Table 3. The absence of WHO ID, FID (1), and IDWA predisposed to intermediate (OR (95%CI): 2.487 (1.512-4.091), 2.447 (1.594-3.758), and 1.928 (1.272-2.955), respectively) and high (OR (95%CI): 2.278 (1.287-4.032), 2.546 (1.551-4.178), and 1.794 (1.111-2.896), respectively) ASCVD risk level.

**Table 3:** Association between iron status and ASCVD risk category. *P<0.05 In all investigated iron deficiency definitions, the presence of iron deficiency and low-risk category were used as reference levels. (+): present; (-): absent; OR (95%CI): odds ratio (95% confidence interval); WHO ID: World Health Organization iron deficiency; FID (1): functional iron deficiency (1); IDWA: iron deficiency without anemia; ASCVD: atherosclerotic cardiovascular disease; low: ASCVD score < 5%; borderline: ASCVD score between 5% and 7.4%; intermediate: ASCVD score between 7.5% and 19.9%; high: ASCVD score > 20%

ID presence		ASCVD risk category		
		Borderline (OR (95%CI))	Intermediate (OR (95%CI))	High (OR (95%CI))
WHO ID	(-)	0.981 (0.484-2.006)	2.487 (1.512-4.091)*	2.278 (1.287-4.032)*
	(+)	-	-	-
FID (1)	(-)	1.294 (0.660-2.537)	2.447 (1.594-3.758)*	2.546 (1.551-4.178)*
	(+)	-	-	-
IDWA	(-)	1.233 (0.623-2.419)	1.928 (1.272-2.955)*	1.794 (1.111-2.896)*
	(+)	-	-	-

The results of MLRA between potential confounding variables and participant iron status are presented in Table 4. Regardless of ID definition, the male gender was negatively associated with ID. Non-Hispanic White participants were more likely to have ID defined as WHO ID and FID (1) (OR (95%CI): 1.987 (1.234-3.199) and 1.718 (1.154-2.557), respectively). Hypertension was positively associated with IDWA (OR (95%CI): 1.934 (1.253-2.985)).

**Table 4:** Association between normal iron level and potential confounding variables. *P<0.05 (+): present; (-): absent; OR (95%CI): odds ratio (95% confidence interval); WHO ID: World Health Organization iron deficiency; FID (1): functional iron deficiency (1); IDWA: iron deficiency without anemia

		WHO ID (OR (95%CI))	FID (1) (OR (95%CI))	IDWA (OR (95%CI))
Men	(+)	0.192 (0.121-0.304)*	0.232 (0.158-0.341)*	0.285 (0.197-0.414)*
	(-)	-	-	-
Non-Hispanic White	(+)	1.987 (1.234-3.199)*	1.718 (1.154-2.557)*	1.116 (0.762-1.634)
	(-)	-	-	-
Hypertension	(+)	0.836 (0.514-1.36)	0.704 (0.461-1.075)	1.934 (1.253-2.985)*
	(-)	-	-	-
Diabetes mellitus	(+)	1.069 (0.550-32.074)	1.430 (0.836-2.444)	1.538 (0.914-2.590)
	(-)	-	-	-

## Discussion

In this cross-sectional study, the absence of WHO ID, FID (1), and IDWA in patients predisposed to increased ASCVD risk. This was a result of the following factors. WHO ID, FID (1), and IDWA utilize certain serum ferritin thresholds in their definition. Increased ferritin levels in groups with higher ASCVD risk could lead to a lower prevalence of ID in patients with low ASCVD risk and give a misleading impression of the “protective effect” of ID status. Patients with elevated ASCVD risk had higher serum ferritin levels and were less likely to be iron deficient. Ferritin levels higher than these ID definitions cutoffs were associated with increased ASCVD risk. Coincidental results were revealed by Masini et al. [12] in a large retrospective study based on patients with HF. Ferritin < 100 ng/mL (FID (1) threshold) was associated with lower cardiovascular mortality and tended to be associated with lower all-cause mortality [12]. Ferritin < 300 ng/mL was associated with both lower all-cause and cardiovascular mortality [12]. Ferritin levels < 100 ng/mL were associated with better survival [12].

TSAT, another iron status marker, was comparable in all analyzed ASCVD risk groups. TSAT reflects the utilized amount of iron, i.e., the relative amount of transferrin that is loaded with iron [26]. Recent studies proved that TSAT< 20% provides the best diagnostic accuracy for bone marrow ID, the gold standard of iron assessment [12,27]. In the analyzed cohort, TSAT levels were higher than 20% and were not statistically different in all ASCVD risk groups. The included ID criteria that rely on serum ferritin have been questioned for their prognostic utility [12]. Ferritin was found to be less useful in assessing ID than TSAT in recent reliable studies [12,27]. It seems reasonable to assume that despite different WHO ID, FID (1), and IDWA prevalence and higher serum ferritin levels in groups with greater ASCVD risk, iron status was comparable in all analyzed ASCVD risk groups.

All these arguments rose a question about current ID criteria utility in non-anemic patients with increased ASCVD risk without cardiovascular history. As the patient’s iron status was assumed to be comparable in all ASCVD risk groups, making an ID diagnosis with the most common definitions could be inaccurate. The employment of ID definitions that only rely on serum ferritin levels could potentially lead to underdiagnosed patients in the high ASCVD risk group and overdiagnosed patients in the low ASCVD risk group. Coincidental observations were made in two large studies based on the HF population [12,28]. ID defined as TSAT < 20% was proposed as a potential solution, as it outperformed WHO ID and FID (1) in all-cause and cardiovascular mortality prediction [12,28]. Accordingly, basic science research questioned the diagnostic value of ferritin and demonstrated that TSAT < 20% had the best performance in selecting patients with ID [27]. The authors suggest that TSAT < 20% could be a better ID indicator in non-anemic patients with increased ASCVD risk without cardiovascular history.

Finally, WHO ID, FID (1), and IDWA were negatively associated with the male gender, which is consistent with results presented in other epidemiological studies [4,5,7]. Males tend to have elevated serum ferritin levels [4,5,7] and thus were more likely to exceed ID thresholds. Non-Hispanic Whites are more likely to have ID due to lower serum ferritin levels, which is in accordance with previous studies on the US population [29]. Only in IDWA definition, hypertension was positively associated with ID. However, the diagnostic algorithm of IDWA encompasses the presence or absence of chronic disease [25]. In this investigation, hypertension was classified as a chronic disease; thus, the determination of iron status could be biased.

This study had multiple limitations. Due to its cross-sectional design, this study did not provide cause-and-effect relationships and had a low level of evidence. NHANES selectively reports the TSAT variable; thus, only records from 2005-2006, 2017-2018, and 2017-2020 were utilized, which significantly decreased the number of participants. The authors did not include new hypothesized definitions of ID in heart failure, TSAT < 20%, and serum iron $\leq$ 13 µmol/L [12,27]. Neither any publication nor any guidelines recommended them for routine use. Finally, the history of cardiovascular diseases was defined by self-report and is vulnerable to both reporting and recall bias. Despite these weaknesses, this evaluated the most common ID definitions on non-anemic patients with increased ASCVD risk without a history of cardiovascular diseases and rose a question about proper ID definition for this population.

## Conclusions

Serum ferritin levels were higher in patients with greater ASCVD risk. Patients with increased ASCVD risk were more likely to exceed ID thresholds. This explained lower ID prevalence in groups with higher ASCVD risk. Due to similar TSAT concentrations, it was assumed that real iron status was comparable in all ASCVD risk groups. The presented differences in ID prevalence, diagnosed with most common ID definitions that rely on serum ferritin thresholds, rose questions about their utility in non-anemic patients with increased ASCVD risk without cardiovascular history. ID definition that utilizes TSAT < 20% cutoff could be a more reliable indicator of iron status in this group of patients.
